# Brackish knowledge: exploring the material, epistemic, and institutional entanglements of numerical modelling of the Dutch coast

**DOI:** 10.1007/s40656-025-00695-1

**Published:** 2025-10-09

**Authors:** Jacqueline Ashkin, Sarah de Rijcke

**Affiliations:** Centre for Science and Technology Studies, Leiden University, Leiden, The Netherlands

**Keywords:** Brackish, Estuary, Ocean, Modelling, Valuation, Research governance

## Abstract

Estuaries, the dynamic transitional zones where rivers converge with oceans, represent complex ecosystems characterized by the mixing of fresh and saltwater, resulting in what is known as brackish water. These coastal interfaces, along with tidal flats and other littoral features, embody a unique duality, existing as neither fully terrestrial nor entirely marine environments. This ambiguous nature poses significant challenges for scientific inquiry when coastal regions become the focus of study. Inspired by Stefan Helmreich’s (2011) call to ‘think with seawater’, we propose the concept of “brackish knowledge” as a way to engage with knowledge practices that are entangled with both the material complexity of the environments they describe and the practical contingencies of the contemporary science system. In this paper, we follow the development and maintenance of the General Estuarine Transport Model (GETM), a hydrodynamic model designed to represent the complex tidal processes in estuaries and shallow shelf-sea areas such as those along the Dutch coast. We show how the model’s developers move across and recombine properties that are often framed in opposition to one another, namely the physical and ecological, social and computational, and public and private, in order to continue making knowledge about coastal and estuarine environments. We conclude that the material, epistemic, and institutional dimensions of brackish knowledge should be considered alongside one another in the governing of scientific knowledge about environmental change, since this ultimately shapes what can be known about potential coastal futures.

## Introduction

On January 31, 1953, the Netherlands experienced the worst flood in its modern history. During an unusually violent North Sea windstorm, millions of litres of saltwater overwhelmed coastal defences in the southwestern province of Zeeland, rushing into agricultural lands and houses alike. The flood killed nearly two thousand people and displaced many more. The aftermath of what is now generally referred to as the *Watersnoodramp* (literally “the flood disaster”) has transformed the landscape of the southern provinces to this day, influencing how the Dutch come to know and manage their coastline (Bijker, 2007; Helmreich, 2023). What started as the Delta Institute for Hydrobiological Research in Zeeland in 1957 is today one part of the Royal Netherlands Institute for Sea Research. It was created in the wake of the 1953 flood and the subsequent decision to implement the Delta Works, a flood defence system consisting of 3 locks, 6 dams, and 5 storm surge barriers (Slomp, 2012). Although the Delta Works would not be completed until 1997, concerns quickly emerged about the ecological impact of the structures that would block the natural flow of salty seawater into the Eastern Scheldt and surrounding estuaries. This is in no small part because this region of the Netherlands has a long history with fishing and aquaculture, dating well back to the medieval period when Zeeland was a series of marshy islands accessible only via the water. It was the task of the newly formed institute to perform scientific inquiries and understand how local fauna and flora were (and would be) affected by the new protective structures that would keep salty water at bay.

By the 1970s, because the fishing and aquaculture industry continued to account for the mainstay of the regional economy, fishers began to protest the complete closure of the Eastern Scheldt, which they saw as synonymous with the closure of their livelihoods. They knew that, sensitive to the salinity levels in their estuarine habitat, many of the fauna the industry relied upon would likely die if the flow of saltwater was cut off entirely. One of their first actions was to cover the new delta institute building with protest slogans, including one that emphasized that the Eastern Scheldt should be left salty (Schipper 2008).^1^ After half a decade of intense debate, politically and beyond, it was eventually decided in 1976 that in the place of a completely closed dam, a storm surge barrier would be built and the coastal defences in the area raised. The storm surge barrier would remain open except in cases of extremely high water levels, when it could be temporarily closed to protect the communities living behind it, but otherwise the flow of saltwater would continue. The task of the delta institute was still to perform scientific inquiries and understand how local fauna and flora were (and would be) affected, but now with a focus on examining the changing flows of salty water rather than its total exclusion.

Between one quarter and one third of the Netherlands is below sea level, and living below sea level does not come without its risks. The Dutch dedicate extensive resources to managing coastal infrastructures, and in recent years have even turned to importing this knowledge to other (especially developing) nations (Dewan, 2021; Ley, 2021). Numerical modelling lies at the heart of this endeavour to know and manage flows of water, proliferating in both public research institutions and the private sector (Jensen, 2020; Landström, 2023). In the post-war period, numerical modelling became a core practice of environmental sciences like climatology and eventually oceanography because of major advances in computing and increasing interest within environmental science disciplines to apprehend something like a global climate (Edwards, 2010; Heymann, 2019; Hulme, 2010). While a majority of early environmental research was highly specific to the places they were conducted, this new turn to global-scale models corresponded with the emergence of environmental governance organizations similarly concerned with the global (Heymann, 2019; Turnhout et al., 2016). During this time, the global scale, and especially global scale numerical models, became the hegemonic way of knowing environmental change. In part this is because of the perceived universality and objectivity of computing practices, built on appeals to rationalism and an accounting culture that implores people to trust in numbers (Porter, 1995; see also: Chan, 2014; boyd & Crawford, 2012; Strathern, 2000; Espeland, 1998).

Theodore Porter (1995) argues that ‘trust in numbers’ is predicated on the expectation that they have been produced through processes of ‘mechanical objectivity’: ‘Strict quantification, through measurement, counting, and calculation, is among the most credible strategies for rendering nature objective’ (Porter, 1995, p. 74). Indeed, the proliferation of numerical modelling in marine research has a strong epistemic dimension. Unable to physically access many areas of the ocean themselves, researchers increasingly rely on automated tools and remote sensing systems in order to know their objects of study, producing vast quantities of data in the process (Lehman, 2018; Turkle, 2009). Computational practices such as numerical modelling play a key role in making these data intelligible, and unlike other forms of scientific experimentation, are capable of addressing uneven data availability to generate insights into unknown or inaccessible dimensions of space and especially time, i.e. the future (Hastrup & Skrydstrup, 2013; Edwards, 2010). Numerical models use mathematical equations to map not only the flow of water, but also the things that flow with it: sediments like sand or nutrients like nitrogen. In this way, models help researchers understand and potentially predict the volatile and changing conditions for life at the coast.

Beyond the epistemic dimension, quantification also serves as a powerful tool for governance: the appeal of numbers grows stronger as the gap between administrative levels and the operational level widens (Porter, 1995). This is because quantification offers a semblance of objectivity and fairness, which becomes increasingly crucial when personal trust and intimate knowledge are lacking. Porter demonstrates that the drive for quantitative rigor is not inherent to scientific activity, but rather emerges from external pressures and the need to establish credibility in contexts where trust is scarce and traditional forms of authority are weak. Thus, numerical practices become a strategy for impersonal decision-making, allowing bureaucracies to manage complex systems and justify their actions through seemingly objective metrics. Porter’s analysis provides a crucial foundation for understanding the trends described in recent literature on academic evaluation and knowledge production. His work connects directly to studies exploring the emergence of new methods for measuring and evaluating academic work since the late 1970s, which have profoundly altered scientific institutions and knowledge production cultures. Research in Science and Technology Studies (STS) and related fields has documented these changes, linking them to broader trends such as the evaluation society (Dahler-Larsen, 2011; Power, 1997), new public management (Burrows, 2012; Shore, 2008), and academic capitalism (Hoffman, 2011; Slaughter & Leslie, 2004). Porter’s insights on quantification as a response to external pressures and a strategy for legitimacy align with this literature. His concept of ‘mechanical objectivity’ through quantitative methods resonates with the focus on expanding evaluation mechanisms in academia, highlighting how institutions use metrics to establish credibility in an increasingly competitive global environment.

Research governance has in the past three to four decades been oriented towards fostering excellence in scientific research, researchers, and institutions (Scholten et al., 2018; Sigl, 2019), with recent shifts also encouraging the development of societally relevant research lines (Hessels et al., 2009; Sigl et al., 2023). These shifts align with and are in many cases the direct consequences of neoliberal governance regimes that expect public institutions to compete for resources on the free market and account for their spending through ranking and rating systems intended to measure quality (Berg et al., 2016; Shore & Wright, 2015). The naturalized common sense behind *audit cultures* (Strathern, 2000) is part of what makes them so pernicious: concepts such as excellence and relevance are as hard to negate as they are to define (Flink & Peter, 2018). Recent studies show the constitutive effects of these policies on research practices, as the measure becomes the goal and researchers are increasingly concerned with journal and individual indicators, grant funding applications, and limited time to dedicate to research (Felt, 2017; Hammarfelt & de Rijcke, 2015; Hammarfelt et al., 2019; Hessels et al., 2019; Müller & de Rijcke, 2017). Limited resources and a scarcity of long-term employment opportunities have intensified competition amongst researchers themselves and effectively transformed them into quasi-entrepreneurial managers of their own careers subject to a system of academic capitalism (Fochler, 2016; Rushforth et al., 2018). In this context for valuing scientific knowledge, excellence and relevance have become the two core registers of worth which determine access to resources, ultimately dictating what knowledge is made and who can make it.

Given these changes in both the making and governing of scientific knowledge, we ask: what happens to scientific knowledge practices that do not fit clearly into the registers around which research governance is organized? What consequences might this have for knowing coastal futures? Bringing together literature from the social studies of water, modelling, and research governance, this paper addresses the ways in which knowledge-making practices are entangled with the complex materiality of environmental assemblages and the material conditions of contemporary research systems. To capture this complexity, we propose the concept of *brackish knowledge*, inspired by Stefan Helmreich’s appeal to think with seawater (2011). This concept draws on the specific properties and qualities of coastal environments like estuaries and tidal flats to reflect on knowledge-making practices that are intimately entangled with the environments they describe but are nevertheless subject to the registers of worth prescribed by research governance. We turn our attention to one model in particular, the General Estuarine Transport Model (GETM), a hydrodynamic model that specializes in describing the physics of estuarine environments. GETM first emerges through scientists’ efforts to understand the changing conditions of the Eastern Scheldt, beginning in the 1990s, and is still in use by coastal ocean researchers today. We follow how this work emerges and why it continues to be possible, demonstrating the importance of attending to when and how scientific labour becomes valuable in the contemporary science system. We conclude that research (e)valuation practices have consequences not just for individual researchers, but also for the environments and communities entangled in the scientific endeavour.

We explore the concept of brackish knowledge by attending to numerical modelling practices ethnographically. We employ assemblage methodologies (Baker & McGuirk, 2017) by combining document analysis, semi-structured interviews, and insights drawn from ethnographic engagements with what is today the Royal Netherlands Institute for Sea Research to present an empirical journey through brackish waters. We analysed a wide range of documents, including materials related to funding calls, research project proposals, and scientific publications and documentation about GETM and other related hydrodynamic models. We used the documents to gain a better understanding of the technical capacities of hydrodynamic models and how researchers argued for the strengths of different modelling approaches, as well as to better grasp the contemporary governance context in which many regional hydrodynamic models emerged in the 1990s. Researcher Ashkin conducted semi-structured interviews with the model’s two core developers and the former leader of the project in which GETM was developed, in addition to interviews with other ocean modelers who used or were familiar with GETM. She also undertook 18 months of ethnographic fieldwork with ocean modelers, consisting of weekly site-visits where she participated in research meetings, joined field excursions, and observed researchers in their daily practices. These engagements inform the ‘ethnographic sensibility’ in our analysis of GETM (Shore & Wright, 2011).

As computational practices increasingly shape our understanding of the ocean, this paper explores how numerical models, specifically the General Estuarine Transport Model (GETM), illuminate this interplay between epistemic objects, epistemic properties, and governance conditions. Below, we explain the concept of brackish knowledge in more detail and introduce our case study, GETM, in the context of advancements in numerical environmental modelling. Next, we will examine how GETM emerged from interdisciplinary collaboration, addressing ecological and physical concerns specific to coastal environments. The model’s development was driven by its unique epistemic properties, particularly its ability to represent the drying and flooding dynamics of tidal flats while adhering to the open-source ethos of the modelling community. Additionally, we will discuss how researchers navigated limited funding for non-quantifiable academic outputs by creatively sustaining the model through resourceful strategies, including founding a private business to support public research. Through these cases, we will demonstrate how GETM’s value is shaped by its capacity to bridge categories such as ‘economic’ and ‘societally relevant,’ offering insights into the broader dynamics of computational ocean research. Using brackish water as a theory machine (Helmreich, 2011) and numerical modelling as a practice through which to chart our course, we analyse how situated knowledge for coastal futures is being made under increasingly pressurized research conditions.

## Brackish knowledge and the case of GETM

Estuaries are aqueous environments where freshwater rivers meet the ocean, resulting in brackish water: water that is neither fresh nor salty, but somehow both. Brackish waters pose intriguing challenges for ecosystems that are often adapted to either fresh or saltwater and can cause serious problems for everything from piping infrastructures to agricultural fields if they flow in unwanted and unanticipated ways. Brackishness occurs in a place of both a confluence—a place where salt and freshwater mix—and an overlap—a place where salt and freshwater sit together. In certain parts of the world, the areas where fresh and saltwater meet are so distinct as to have a clear boundary line, with murky brown freshwater on one side and clear blue seawater on the other. The illusion that they do not mix, however, is just that—an illusion. Influenced by temperature, tides, and currents, the fresh and salt water mix together along a narrow gradient. The trick is that this gradient is not perpendicular to the seafloor. Since saltwater is denser than its fresh counterpart, it sinks and forms a distinct layer underneath, one that can travel for kilometres inland. In estuaries these confluences and overlaps matter in part because as freshwater mixes with saltwater, the water’s ability to carry sediment transforms. As the waters mix, salt ions bind to the floating sediments and they sink, creating a new layer on the sea floor. Over time, brackish waters bear the potential to build land by depositing sediment along the Dutch coast, or to erode it away, carrying sediment out with the tides and into the North Sea.^2^ As such, understanding how water flows through estuaries has consequences for planning and preparing coastal futures (Ley & Harms, forthcoming).

Much like the mixing of fresh and saltwater in estuarine environments, brackish knowledge offers a way to understand scientific knowledge-making practices that traverse established categories and relations. Brackish knowledge is a way to understand scientific knowledge practices that travel “athwart” established categories of and relations between (Helmreich, 2011). It resists binary thinking and instead focuses on the complex interplay between different forms of knowledge, methodologies, and empirical realities. This concept encourages us to trace the contingent, drifting, and often unexpected connections that constitute social action in scientific research, particularly in the context of environmental modelling. Brackish knowledge builds on the insight from field-based science studies that knowledge practices are entangled with the environments they describe (Brinitzer & Benson, 2022; De Bont, 2009; Kohler, 2002), while incorporating the equally constitutive dimensions of research governance. This approach is particularly relevant when examining numerical models like GETM, which attempt to capture the intricate dynamics of coastal systems while navigating the demands of academic evaluation and research governance.

Conceptually, the term ‘brackish knowledge’ builds on a long tradition of scholarship in science and technology studies which point to the situated nature (Haraway, 1988) and often ‘messy shapes’ (Wouters et al., 2008) of knowledge making, insisting that material, epistemic, and institutional dimensions must always be considered together.^3^ By following the genesis and maintenance of one hydrodynamic model in particular, we show how brackish knowledge emerges through the muddy entanglement of epistemic objects (the physical or tangible aspects of the research, such as the coastal environments, water flows, and sediments that are being studied), epistemic properties (the methods and processes of knowledge production, such as the use of numerical modelling and quantification techniques), and governance conditions (the institutional and administrative contexts in which research is conducted and evaluated). In each of these dimensions, our interlocutors work athwart dominant oppositions, weaving together the physical and ecological, social and computational, and public and private elements in their efforts to make scientific knowledge about coastal estuaries. We propose brackish knowledge as a sensitizing concept that helps attend to the specificities of knowledge practices that might otherwise be overlooked in existing governance strategies, especially given the widespread trust in numbers generated by and about environmental models.

The General Estuarine Transport Model, otherwise known as GETM, serves as a prime case study of how numerical modelling has become integral to coastal research, particularly in the Netherlands’ ongoing efforts to understand and control water flows. GETM is a hydrodynamic model that exemplifies the trust placed in numbers and computational methods to know and eventually manage complex environmental systems. By simulating the movement of water and sediment through coastal areas, GETM embodies the principles of mechanical objectivity that Theodore Porter (1995) describes. It divides the ocean into grids, solving mathematical equations for each section to produce data that can be visualized as tables, charts, and maps. GETM is a model especially suited to understanding processes in coastal and shelf seas, areas that are significantly shallower than the open ocean and therefore requiring dedicated attention to physical processes deemed inconsequential or irrelevant in models of the global ocean. Originally developed to simulate the Eastern Scheldt at the turn of the twenty-first century, the model is still in use today to research coastal areas, especially around the North and Baltic Seas. GETM is a particularly fruitful case because of its original focus on estuaries, environments which also straddle categories often held in opposition to one another such as fresh versus saltwater, wet versus dry, and local versus global.

Advancements in computing have transformed the study of environmental change dramatically, especially in recent years as technologies have become increasingly accessible. In his history of atmospheric climate modelling, Paul Edwards describes weather forecasting as “the first computational science”, emerging in the wake of computational advancements following World War II. The timing was not accidental—predicting the weather was a practice born of military needs, since weather conditions could easily determine the outcome of a battle (Edwards, 2010, see especially chapter 6). Before this technology, calculations to ensure optimal tidal and weather conditions such as those for the Normandy landing were done by hand, using a combination of tide tables, physics equations, and any available data about the specific coastline in question; perhaps unsurprisingly, these methods were overwhelmingly prone to error and often failed to account for weather patterns on larger time scales (Parker, 2010). Predicting the weather eventually gave way to more ambitious models of the global atmosphere, paving the way for the global circulation models that now inform global environmental assessments like the International Panel for Climate Change (IPCC). In this vein, American oceanographers Kirk Bryan and Michael Cox are credited with producing the first ocean circulation model in 1967, although more dedicated development of ocean models would not begin in earnest until the 1990s and would largely focus on representing the global ocean (Griffies et al., 2015).

Models concerned with regional or local complexity are not inherently better or worse than those oriented towards the global scale, but their increased resolution—that is, the smaller size of the grid they are able to model—allow them to include physical features closer to the coastline, such as tidal flats, allowing them to address different types of concerns about environmental change. GETM, for example, can function at a resolution as detailed as a hundred meters at a time, significantly more detailed than global models that may only be able to generate output about one hundred kilometres of coast at a time. At the global scale, the impact of estuaries is lost. As anthropologist Anna Tsing points out in her theory of non-scalability (2012), it is not that the non-scalable is better than the scalable and should be valued as such, but that non-scalability insists on the relationalities that the scalable—in this case, the globalized—erase, and deserve their own attention. Indeed the generation of global models is tightly linked to the needs of global environmental governance institutions such as the IPCC (Heymann, 2019; Hulme, 2010; Turnhout et al., 2016). Efforts to erase geographical difference ultimately generate global forms of scientific knowledge which are less able to contribute to located concerns.^4^ Given the present hegemony of global environmental models, the concept of brackish knowledge guides attention to when different institutional frameworks might be needed to facilitate knowledge practices that can adequately encompass (especially material) specificity.

Physics models like GETM are only one type of numerical model appearing in contemporary ocean research. While the models discussed thus far are by-and-large based on physics equations, numerical ecological models emerge from a different teleology attempting to describe population dynamics using mathematical equations. From its inception, ecology was a largely descriptive and qualitative science quite different from its disciplinary peers and highly dependent on environmental settings (De Bont, 2009; Hackett et al., 2008; Kohler, 2002). It was not until well into the twentieth century that strands of ecology more interested in the dynamics of entire ecosystems began to explore the possibility of describing these dynamics mathematically (Breckling et al., 2011). This was not without reason—the amalgamation of species and processes that together form ecosystems are extremely complex and remain to this day challenging to observe and quantify in a representative manner (Vermeulen, 2013). In ocean research, ecological models tend to focus on biogeochemistry, or the flow of chemical elements through living organisms and environments. Their mathematical equations focus on describing the population dynamics of lower trophic level organisms like phytoplankton and their impact on processes of primary production, tracking the flow of nutrients like carbon and nitrogen. Biogeochemical models are crucial for understanding and predicting phenomena ranging from harmful algae blooms to the potentials of carbon sinking but rely on coupling with physics models to illustrate their spatial dynamics. As a physics model, GETM is an interesting case because of its ability to relate well to both biogeochemical models and the detailed physical dynamics of the Dutch coast. When the initial version of GETM was being developed towards the end of the millennium, it was scientifically novel and part of a still nascent ocean modelling community.

The modelers that we work with do not approach their work with the kind of totalizing universalist zeal that in retrospect seems commonplace amongst many early developers of (especially atmospheric) models. Recent literature in the social sciences have been increasingly critical of numerical modelling practices, their relation to reality, and their potential constitutive effects (Pilkey & Pilkey-Jarvis, 2007; Puy & Saltelli, 2022; Saltelli et al., 2020; Thompson, 2022). In-depth studies of numerical models used for understanding coastal change in particular have shown the ways these models are not as universally applicable as they initially seem (Barnes, 2016; Ley, 2021; Vaughn, 2017). Underlying these critiques is a warning against treating models as reality when using them to make decisions about the world “out there”, especially when model outputs are presented as the singular representation of the world on which to act upon at the expense of other ways of knowing. Our interlocutors are aware of the limitations of their models and emphasize instead that models are useful tools for representation because they can offer insights that encompass time periods or physical geographies that are otherwise beyond the reach of scientific knowledge (see also Hastrup & Skrydstrup, 2013). For modelers, good models are models that are as representative as possible given the social and institutional circumstances in which they find themselves, since representativeness is an ongoing negotiation throughout the development process depending on conditions such as data availability, computing time, and research funding (Ashkin, forthcoming). Critiques about the relationship between reality and representation in numerical modelling are more closely tied to how models circulate than to their use in scientific research, which is the main focus of this paper.

## The material: estuaries and brackish problems

Estuaries trouble existing divisions of labour, which in water research and governance are often divided between fresh and saltwater environments. The water in estuaries is at times fresh, at times salty. Estuaries displace logics of mechanical objectivity, which take water ‘to be a self-evidently “global” substance’ (Helmreich, 2011, p. 137) and allow instead for scientific understandings that attend to the specificities of estuaries and their unique material constitutions of flora, fauna, sediments, and water. Researchers can compare estuaries not because they are identical to one another but because they are analogous in important ways. The North Sea Flood of 1953 made the material vulnerabilities of living by the coast abundantly clear, but as the Delta Works were erected, determining how to live with these vulnerabilities quickly became a source of contestation in Zeeland. The Delta Works were an attempt at taming the as-yet unpredictable flow of seawater, which was as much a source of destruction as a necessary condition for liveliness in the Eastern Scheldt. Brackish problems began to emerge for scientists trying to think about how the newly implemented flood control infrastructures would change the estuary’s physics—how (salt)water flowed—as well as its ecology—what could survive, given the change in water flows. The material contingencies of the estuary came to shape researchers’ epistemic practices, because in order to use models to explore these changes, researchers had to work athwart the disciplines of ecology and physics.

Construction on the Delta Works lasted well into the 1990s. The engineers who constructed the flood control system paid homage to the life in the estuary through the construction process, naming the specially built working vessels *Mytilus, Cadium, Ostrea* and *Macoma,* these being the genus names in binomial nomenclature for local species of mussels, cockles, oysters, and clams respectively. Still, the completion of the storm surge barrier in 1986 did not end centuries of relating to and with the unruly flows of seawater. The barrier changed the way saltwater and sediment moved through the estuary, with consequences for the mussels, cockles, oysters, and clams, creatures which all rely on the flows of water to bring them their food and leave sediment behind in which to make their homes. The fishing and aquaculture industry, who at the time still adhered to established longstanding assumptions in the fisheries management community that marine resources were endlessly exploitable, carried on with business as usual (Finley, 2011; Rozwadowski, 2002; Telesca, 2020). Following advancements in computational tools for scientific ways of knowing, especially following the Cold War (Edwards, 2010), researchers at the delta institute began to explore numerically modelling the flows of water in the estuary as one possible way in which to understand—and potentially predict—the changing conditions for a continued brackish existence.

Peter Herman was one of these researchers, arriving in Zeeland shortly after completing his PhD in estuarine ecology in the late 1980s. The delta institute would become part of the Netherlands Institute of Ecology (NIOO) in 1992. When Herman received his education as an ecologist in the 1980s, very little was known about what scientists refer to as the benthos, creatures that live along or in the sea floor—the mussels, clams, oysters, and other creatures that have featured in our story thus far. Herman is now a seasoned and respected figure in estuarine ecology, having dedicated much of his career to understanding how physical and ecological components interact and developing numerical models about these interactions. He now works as a professor in Delft. JA caught him online between classes, where he told her about the context in which GETM emerged, as part of a large European Union funded project that brought together experts from across the region. “If you are a mussel,” he asked, “and you have to choose your location within an estuary like the Oosterschelde, where would you prefer to live?”.

He began by explaining why hydrodynamics—the way water flows—is so important to creatures like mussels. Mussels are passive feeders, meaning that they rely on the movement of water to bring their food sources to them. Herman and his ecologist colleagues hypothesized that vertical mixing, or the way water flows vertically between the sea’s surface and the seafloor, played an essential role in making certain parts of the Eastern Scheldt livable for mussels. He explained it as follows:

If you only look at … a single location, you have your primary production, the growth of the algae is taking place in the top of the water column. The animals are sitting at the bottom of the water column, and especially if your water column is deep, you need some time to mix in the production [the algae] towards the consumption [the mussels] side. (interview April 3, 2023)

It is from here that the collaboration with GETM’s initial developer, Hans Burchard, first emerges. Burchard was still a young researcher when he met Herman, still fresh from completing his doctoral degree in physical oceanography in Germany. He was doing a postdoc in Italy at the time, but met Herman at a project meeting in France. Burchard and his colleague Karsten Bolding had already developed a different model, GOTM (General Ocean Turbulence Model, see Burchard et al., 1999), that was specifically designed to study vertical mixing in the water column in one dimension. Because mussels rely on currents and tides to bring their food to them, however, it quickly became clear that choosing where to live as a mussel was a three-dimensional problem in need of a three-dimensional tool to find the solution. While GETM is a physics model, it needed certain capabilities, such as the ability to model three dimensions, so that it could more easily be coupled to an ecosystem model about mussels. Developing a new model was an attempt to work athwart disciplines and think ecological and physical concerns together.

Now a professor in his own right, Burchard explains that model development and model application go hand-in-hand. Perhaps unsurprisingly, models require adjustments or additions in order to address the material specificities of different coastal environments. Using an example from a more recent project modelling coastal fjords, Burchard extrapolates:

You have to tell that to the model [about these differences] somehow, and of course the model doesn’t know that, and we have to implement that capability into the model that it can do that. And that’s the latest thing, for example. It’s nothing which is necessarily from our direct region here. Obviously it’s not. (interview November 12, 2021)

Location and *located,* then, is an important distinction for models like GETM. Having access to the code of the model becomes crucial for extending its application and locating it in the materiality of the environment in question. While it is possible to disentangle the development and application of a model—many models work this way—it would.

take a very, very long time to extend the model to the applications we want. And then the question would also be, I mean, if those developments we would do then would actually go back into the master version of that model. (interview November 12, 2021)

Fragmentation is an ongoing concern for models that function open-source, with some additions never being reintegrated into the master version or resulting in such a drastic transformation that an entirely distinct model emerges. When deciding to build a model, material and epistemic concerns overlap and flow together.

Brackish knowledge is characterized by these categorical confluences and coincidences. The geographical location of the institute made the brackish lifeworlds of mussels visible to the researchers at a crucial point in time, elsewhere along the Dutch coast. Although Herman was adamant that the fishing industry was not involved with the European Union funded project through which the model eventually emerged, emphasizing that they did not contribute financially to the research, he also pointed out that.

it was not entirely coincidental that we were also interested in mussels because there were so many mussels and mussels were all around us. […] At the same time, you also had all these controversies in the Wadden Sea on the protection of the mussel beds. (interview April 3, 2023)

In the late 1990s, mussels were doing badly in the Wadden Sea, an intertidal area along the northernmost coast of the Netherlands. No one knew what was happening or why. Although not the same coast, its brackishness provided an important condition for locating knowledge making not by scaling up but by scaling athwart. Rather than attempting to model the entire Dutch coastline, from the Eastern Scheldt to the Wadden Sea, understanding processes situated in the Eastern Scheldt allowed researchers to speak to concerns about other, equally situated phenomena. Brackish knowledge is not an accomplishment of infinite scalability, a project wherein ‘the small is encompassed neatly by the large’ and isolated from relations so that ‘both are crafted for uniform expansion’ (Tsing, 2012, p. 507). Because GETM describes processes specific to estuarine environments, it cannot be neatly encompassed as one unit of a global ocean model, but it can be used to understand other, materially analogous contexts. Brackish knowledge does not strive to be equally applicable in all places and at all times, but this does not mean it is entirely unscalable. It can be scaled laterally to engage with analogous conditions, in this case becoming relevant to brackish problems in other estuarine environments. By attending to the ways material relationalities, to other beings and to the environment, influence the scalability of knowledge practices, governance initiatives might foster insights that scale laterally rather than focus on priming interventions for uniform vertical expansion.

## The epistemic properties of brackish solutions

There are hundreds of hydrodynamic models in existence. The need to develop a new numerical model emerges when figuring epistemic considerations alongside each other which then render existing models inappropriate for the problem at hand. In her work on freshwater modelling, Catharina Landström (2023) distinguishes between models produced in scientific institutions and those used in water management; the overarching goals of each type of modelling differ, since models developed by scientists generally aim for better representations of physical processes while models used by managers prioritize convenience and intelligibility to non-modelling expert users. In both contexts, restricted time and finances leave few resources to dedicate to developing and maintaining efficient code. The decision to develop a new model, then, is based on a number of criteria that reflect what researchers value about the models they work with. As a scientific model, GETM was developed to improve representation by including both ecological and physical factors. However, this required the model to encompass multiple epistemic properties simultaneously. As Casper Bruun Jensen (2020) observes of the plethora of hydrodynamic models in the Mekong delta, the justification for the development of new models is often done through the work of comparison. What is particularly interesting for brackish knowledge is the types of epistemic properties that are employed in the task of comparison, and the ways that they are engaged alongside each other. The epistemic properties that we discuss, namely appropriateness for the case and accessibility, are most noteworthy in the ways that they flow across and into each other. These epistemic properties are characterized by their brackishness: how they are messy, muddy, seeping into each other in such a way that they must be placed in heterarchal relation (c.f. Stark, 2009) to one another because they cannot be disentangled.

**Fig. 1:**
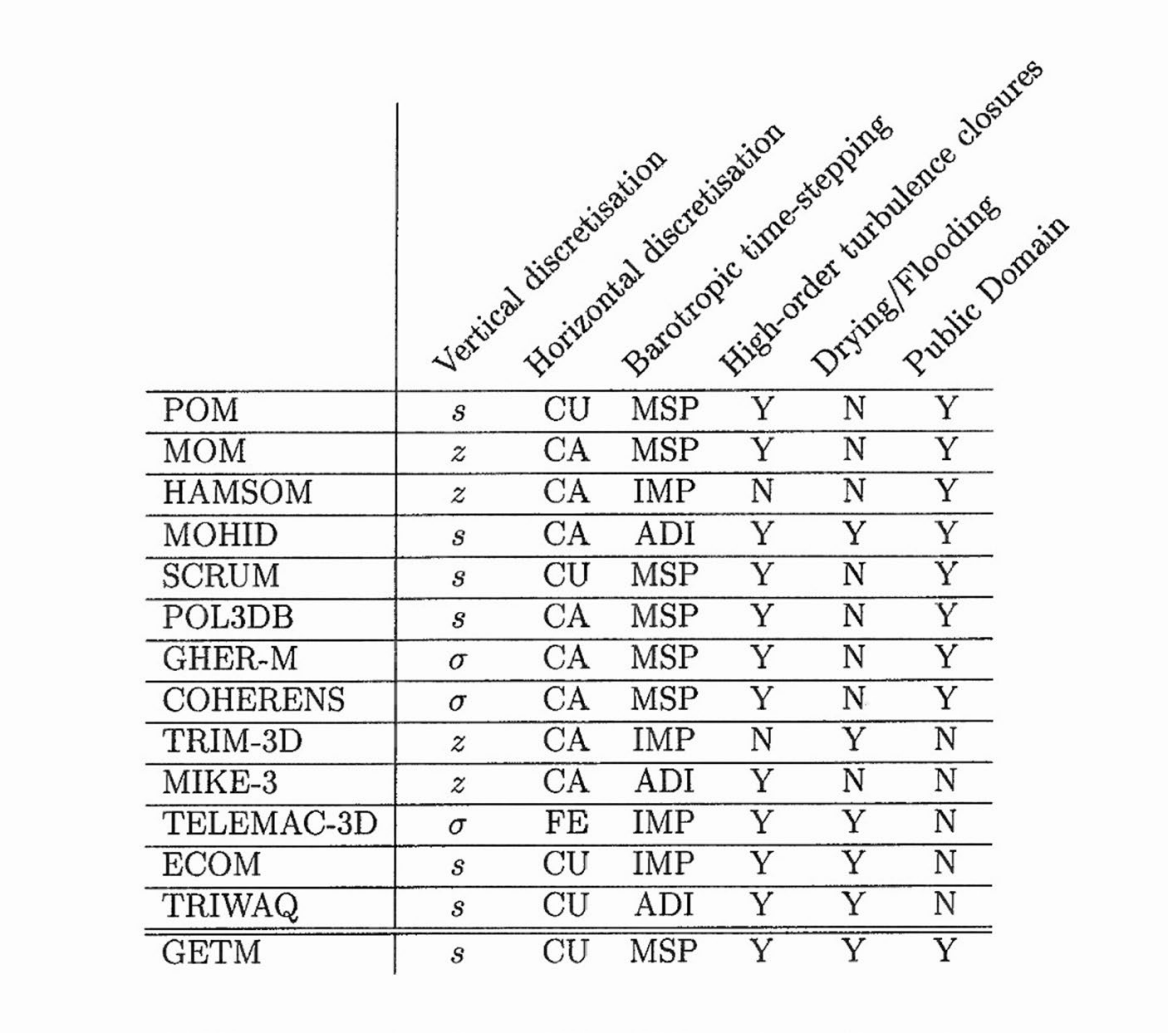
A table comparing a number of hydrodynamic models available at the time. Note the ways that epistemic and organizational concerns sit alongside each other. From the scientific documentation of GETM (Burchard & Bolding 2002, p. 8)

This is best shown by a table that appears in the model’s scientific documentation, used by the authors to succinctly explain their motivation to build a new model (see Fig. 1). The mussels do not make it into this document, but another set of evaluative concerns emerge as the authors justify their addition to the proliferation of hydrodynamic models. On one axis, they list a variety of models available at the time, developed at public research institutions in Europe and the United States. Most of these models are still in use today, and a majority have since become open source. On the other axis, they list the criteria that are relevant to consider when choosing a model. What struck us most about this table was the last column: is the model in the public domain? These criteria caught us off guard considering the highly technical criteria that precede it: barotropic time-stepping, turbulence closures. The table brings concerns about epistemic and organizational properties of the model alongside each other; we expand especially on the criteria in the last two columns, drying/flooding and public domain, because these are also the criteria our interlocutors reiterated in our conversations. They highlight considerations about appropriateness and accessibility that cannot be disentangled when evaluating which model to use for the case at hand.

The most straightforward of the criteria, appropriateness for the case, is the direct result of *literal* brackishness, that is the complex physical geography of estuaries. The Eastern Scheldt is heavily influenced by the tides, creating what is known as an intertidal area, an area that is flooded at high tide and exposed at low tide. During low tide, the water recedes to reveal the dark jagged forms of mussel beds scattered intermittently along the shore. To learn why mussels choose to live where they do, one must know both which resources are available to them and which are not, when they are available and when they are not. At the time, very few hydrodynamic models were equipped to handle the complex numerical equations that could account for flooded and dry areas. As readers might recall from high school math classes, entering zero into a multiplication problem produces another zero as its answer. A similar logic applies here. Entering a null amount of water—a zero—into the equations produced failed results or models that simply would not run at all. The inability to address drying and flooding quickly ruled out a large portion of existing models. These considerations bring concerns about model code and its capabilities alongside the features of the natural environment in question in order to establish whether the model is appropriate for the case or not.

The issue of drying and flooding did not rule out all options—still a handful were left over. But of those models that could account for drying and flooding, all failed to meet the next criteria: accessibility. None of these models were available in the public domain and exorbitant fees made them more or less inaccessible. But there is also a secondary factor at play here: many of the scientists working in this community were well ahead of the curve in regards to developing publicly available and openly accessible software. This is not only a question of limited resources; using a closed model –both in terms of cost and code—went against their ethos towards making scientific knowledge. Part of what made these models so unaffordable was the opacity of the code. If a model is not accessible on the public domain, researchers are reliant on the developers to make any changes or additions to the code. The fees to not only access the code, but have it customized or adjusted, quickly skyrocket well out of the budget of publicly funded research (somewhat ironically, considering many of these models are owned by other research institutions, which in turn struggle to structurally fund model development and maintenance). Adding to the frustration, the desired changes or additions are often adjustments modelers embedded in the research can address themselves if they have access to the original source code and the software in which it is created. Just as fresh and salty water mixes together in an estuary, different types of concerns around cost and code mix together to form brackish criteria for assessing existing models against the potential development of a new one. Researchers bring social and computational dimensions into conversation with one another when making epistemic choices. With no existing models able to encompass these epistemic properties, the brackish solution was to build a new one that did.

## Institutional conditions and keeping brackishness going

After brackish knowledge emerges, it can struggle to stay afloat. This is not unique to numerical modelling practices: across fields, researchers find themselves in a perpetual race to acquire and accumulate (especially financial) resources in a phenomenon described by Maximilian Fochler as ‘epistemic capitalism’, whereby research governance directives have transformed researchers into ‘entrepreneurial managers of their own careers’ (2016, p. 924). For example, Rushforth et al. (2018) show that in the face of precarious and increasingly entrepreneurial neoliberal academic conditions, biomedical researchers in the Netherlands strove to generate *portfolios of worth* containing potential research projects that could appeal to either register depending on the opportunities that were available to them. Portfolios of worth draw on and are a prime example of what economic sociologist David Stark refers to as a *heterarchy*, an organizational form in which there is ‘no hierarchical ordering of the competing evaluative principles’ (2009, p. 19). Stark points to flexible organizational forms that enable members to participate in activities that appeal to registers of value outside of the dominant registers in those organizations. Many of the ocean researchers we work with feel they have no choice but to engage if they want to continue their work, and modelers are no exception. Once a model has been developed, a new set of challenges emerge around its maintenance. A numerical model, like any other scientific infrastructure, demands resources in order to remain useable and useful for knowledge making. While developing a new model is often considered novel and cutting-edge, and therefore eligible for excellence funding, the same cannot generally be said for the labour involved in maintaining a model. This is part of a wider culture that values innovation over the humdrum, everyday work of maintenance, which Russell and Vinsel (2018) point out is a form of labour that is regularly overlooked. Emerging from the mixing of existing registers, brackish knowledge may not immediately become institutionalized because there is no straightforward niche for this form of labour.

In the case of numerical modelling, maintenance labour is rarely recognized or supported institutionally. The costs of maintenance, financial or otherwise, are instead passed on to individual researchers who must compile consortia or ad-hoc support so that their models continue to be useable. Tasks involved in maintaining a model include improving code to quicken runtimes, updating code to run on new software, and adding new processes -i.e. keep pace with the newest scientific findings—so that the code better represents the coastal environment or more easily addresses specific scientific topics of inquiry. In some cases, consortia form around specific models and are able to combine infrastructural funding with small injections of money from individual researchers’ own sources. This strategy is occasionally able to account for the manpower necessary to consistently perform maintenance tasks, as research institutions, already pressed for finances, often do not have the resources or the structural capability to take on staff dedicated to maintaining code without actively participating in scientific knowledge making. Maintenance requirements push researchers into brackish territory, where they must think athwart these features of the contemporary science system to patchwork resources together and keep their model afloat.

Numerical modelling is a practice that requires an immense amount of technical expertise for coding in languages such as R, Python, and FORTRAN. Learning to code is often likened to learning a new language, and just as with prose, not all code is written to the same standard. While it may sound rather obvious, first iterations of model code are already considered successful if the model functions for the purpose intended and generally fits with available observational data.^5^ Imagine this model as a car with no suspension and the doors duct-taped on—it will still run, but not as well as it could. Well-written code saves computing time (and therefore resources) because it is easier for (super)computers to process and streamlines the incorporation of updates about the physical processes described, making it easier to maintain over time. Coding platforms and languages also change over time, meaning that for example older code written for FORTRAN77 may need to be significantly overhauled in order to function with later versions of Fortran e.g. FORTRAN03 or with other coding languages that gain popularity over time, e.g. C or Python.

Hans Burchard’s colleague Karsten Bolding is a talented software developer and spent much of his early collaborations with Burchard refining the code of both GOTM and GETM. He still spends much of his time in the depths of model code, often rewriting or adding new features. When JA spoke to him, he told me about current efforts to transition GETM away from any one specific coding platform so that it would be easier for users to access regardless of their computer operating system. This is not just a case of obsolete coding language, since most young researchers are now trained to use Python rather than FORTRAN; rewriting the code makes ‘the entire process and the entire program more simple and more transparent, so it would be easier for others in the future to contribute’. Generating and improving code is at the heart of maintaining a model, not least because it opens up opportunities for relation.

The problem being that generating and improving model code, in and of itself, is not a labour that is rewarded in the context of a research system increasingly geared towards publication outputs and grant income (Franssen et al., 2018; Hammarfelt & de Rijcke, 2015; Sigl et al., 2020). The developers emphasized the need for staff whose time could be dedicated to coding rather than being divided amongst the myriad other tasks expected of researchers such as writing papers or supervising students. Finding a way to fund such a position proved difficult for multiple reasons: the expertise for such a position requires not only an advanced knowledge of coding languages, but also an in-depth knowledge of the physics of the ocean, generally at a doctoral level; once in such a position, it would be difficult for the expert in question to progress to another research position, given their lack of publication and grants; and of course, with public research institutions increasingly pressed for funding availability, the financial resources to make such a position permanent are simply unavailable. This is all without considering the need for an overlap, if not an alignment, between the values and priorities of the system with those of the individual researcher who might take on such a role.

Given these institutional and systemic constraints, the developers opted to form a business through which to fund the roles and expertise that did not fit in institutional research contexts. Bolding now runs this business side of GETM alongside another colleague. He tells JA that early on his career he decided he was not particularly interested in publishing:

I don’t like writing too much. I’m always sort of developing [model code]. That’s sort of where I find pleasure in it. But [with the business] our career is not dependent on publications. It does not mean that we do not publish every now and then, but it’s not very often. But then when we do, it’s very often cited. (interview November 24, 2021)

This builds on a larger observation that JA heard from many modelers, connecting institutional publication pressure to their potential career trajectories. Modelers like Bolding value their autonomy from institutional routines while nevertheless caring deeply about the progress of their research, publishing as and when they feel they have important contributions. While this would not do for an academic career, Bolding is pleased that he ‘still managed to survive’ working on code by means of the private business.

Rather than appealing to capitalist logics of private commodities, however, the model has remained explicitly open source and available to the public. The developers describe the business as an opportunity for more flexibility and research freedom, with Bolding emphasizing that ‘none of us are interested in business whatsoever. We are interested in having our freedom to do what we can, what we like and what we are good at’ (interview November 24, 2021). Their website goes into further detail, pointing out that:

Over the last years it has been increasingly difficult to obtain sufficient funding to carry out our contribution to the further development of the various software projects we are involved in—most notable GETM and GOTM. As a consequence we will try out a ‘crowd funding’ approach where we will try to obtain the funding of our contributions through financial contribution from the users of the software. The idea is that we develop a new feature (either on our own initiative or through the request from others) and—when—in a state where it is ready for beta-release we announce the software on this site together with an amount to cover our development expenses. In case sufficient funds can be raised the new feature will be released to the public via the public git repository.

The notion of a private business to fund public research points to the brackishness of these knowledge making practices and how they consistently straddle multiple registers of value. The developers argue that the openness of the model has played a crucial role in its survival, even before making code openly available was a common practice, because it allows for users to experiment with the model and see where there are opportunities for potential code development. In this way, rather than going in search of customers, generating income relied on the developers’ existing networks of colleagues approaching them and saying “ ‘we tried it out and we would like to have this added, can you add that and then we’ll pay you a bit of money for that’.” Burchard eventually stepped away from the company because of his permanent position in academia; by “leaving” the company, it was easier to become a “customer” and pay for the development work of his colleagues. In brackish knowledge there are no neat delineations between seller and buyer or private and public commodity. The persistence of the model relies on its valuation through relations that sit athwart traditional understandings of these categories and their resulting practices.

This is of course a precarious line to ride. The main challenge to the model looking forward was invariably funding: when people no longer need developments, the money dries up. Turning to a private business model is also not particularly scalable in the long-term, in part because of the project-by-project nature of generating income and in part because even when there are talented students with the right combination of computational and environmental expertise, there are no long-term positions available. Additionally, while they pointed to the opportunities offered by working athwart institutional contexts, developers adjusted their day-to-day modelling practices to compensate for the kinds of infrastructures they no longer had such ready access to. For example, without access to large computing network, they worked so that code could be checked on standard at-home computers before being sent to research partners who could afford processing time on a supercomputer. Or bypassing these complex institutional networks, they rented virtual computing space from the Amazon Elastic Cloud. Bolding and his colleague now have enough projects to work on the business side full time without relying as heavily on the crowd-sourcing approach, and they maintain a tight collaboration with Burchard and other academic researchers. Despite their lack of interest in “doing business”, the developers find themselves engaged simultaneously in multiple modes of capitalist relation—whether those within research institutions (Fochler, 2016) or within the wider market economy—in order to produce brackish knowledge.

## Conclusion: brackish knowledge

As the history of the Eastern Scheldt has shown, brackishness is contested as a condition for existence – both for humans and for other life forms. It is not uniformly distributed, and neither are its effects. Brackish water is not water out of place, although it can be when it forces its way past coastal defences or ventures inland to corrode piping and upset agricultural crops. Instead, brackish water forces attention to relations that are tidal in nature, temporary and contingent. It offers chances at flourishing as much as it may take them away. Brackish water is good to think with because it is water in relation to itself, but also to the lifeworlds that inhabit and rely on it. Looking especially to the brackish waters that wind their way through the estuaries, deltas, and marshes of the Dutch coastline, this paper has explored the affordances of reorienting towards the aqueous, the watery, and the partially amphibious for research governance in the context of climate crisis.

Brackish knowledge, then, allows us to think with the materiality of water to better understand and value the abundance of ways to make knowledge for life on a changing coastline. It is an attempt to think the materiality of complex coastal environments together with the material contingencies of doing scientific research. As computational practices come to play an increasingly crucial role in knowing the ocean, numerical models provide a unique doorway into understanding the confluence between epistemic objects, epistemic properties, and governance conditions. GETM was born out of interdisciplinary collaboration and the need to incorporate concerns from both ecology and physics specific to the epistemic object (read: coast) in question. The modelers identified a unique combination of epistemic properties in the model that made it worthwhile to develop, namely that it was capable of representing the drying and flooding characteristic of tidal flats while still being open source and true to the ethic of the modelling community. Amidst scarce funding for academic labour that does not produce countable outputs such as publications, the researchers wove resources together to maintain the model, starting a private business aiming to support their public research. In each of these moments, the model comes to be valuable through the work of combining and moving across categories such as ‘the economic’ or ‘the societally relevant’.

GETM is just one example of how modelers expend significant time and energy to strategically navigate research systems in order to produce valuable knowledge about situated coastal environments. It demonstrates the ways in which a lack of alignment with institutional or systemic values does not necessarily leave particular lines of research undone or irrelevant. The developers did not disentangle from the research system entirely; nor did they fully embrace free-market principles in their entrepreneurial endeavours. According to other researchers who use the model, GETM continues to be one of the best models for studying tidal flats and estuaries like those along the coasts of Germany and the Netherlands, ecosystems which provide a vital buffer in the increasingly probable event of environmental catastrophe. Brackish knowledge emerges from muddy conditions: materially, epistemically, and institutionally. These conditions are not quite fresh, not quite salty, but always both together. Thinking with water displaces objectivist scientific descriptions of water that take it ‘to be a self-evidently “global” substance’ (Helmreich, 2011, p. 137) and allow instead for alternative understandings that attend to the specificities of located waters, as well as their entanglements with and their implications for the making of scientific knowledge. Producing lines of research that do not fit neatly into preexisting categories or registers of worth assumed by research governance strategies relies on the time and efforts of a handful of experts to work *athwart* the systems they participate in, piecing together resources to keep making brackish knowledges (possible).

Much existing scholarship of numerical modelling remains highly sceptical of its attempts to capture the complexity of entire systems (Morita & Suzuki, 2019; see also Thompson, 2022; Puy & Saltelli, 2022). In their article on the patchy Anthropocene, Tsing and colleagues (2019) point to the limitations of knowledge framed in global terms by highlighting the importance of noticing landscapes and the particular, located entanglements of environmental change. As we have shown, modelers of coasts and estuaries acknowledge particularity and locatedness as key characteristics of their field. Reflecting on the future of coastal and estuarine models, colleagues of our interlocutors emphasized the importance of developing and maintaining a wide array of models that can approach different coastal conditions in all their specificities (Fringer et al., 2019). Such knowledge making is only possible with careful attention to the messiness of scientific practice and the contingencies researchers are subject to in the process. ‘Learning to think structures and systems differently’, then, is also learning to rethink the networks and alliances that come to constitute knowledge of environmental change (Tsing et al., 2019, p. S187). A patchy Anthropocene requires corresponding patchworks of knowledge making, resistant to projects of infinite scalability and attentive as much to the similarities of coastal environments as to their differences.

Here we have focused on scientific knowledge practices, and numerical modelling in particular, showing that although scientific knowledge is often valued at the top of the hierarchy in relation to other ways of knowing, science governance imperatives generate new hierarchies within and amongst research institutions by determining who can access resources and on what terms through competitions that value some labours and knowledges over others. As Whitley and colleagues (2018) write, assigning resources to research according to narrowly defined objectives ultimately reduces the diversity of research strategies and practices. Brackish knowledge builds on this observation, adding that valuing research is not just a question of epistemic innovation but has active consequences for the environments and communities entangled in the knowledge making endeavour. If the assumption of research governance is that it can—and indeed, should—steer research in directions more explicitly oriented towards environmental futures, brackish knowledge points to how “science finds a way”, as one of our interlocutors so succinctly phrased it. By exploring the story of one model and the relatively privileged group of researchers who develop it, brackish knowledge asks us to consider who can afford the extra labour that ‘finding a way’ entails and what consequences this might have for researchers with fewer institutional resources at their disposal. What ways of knowing could be possible if registers of value are given the opportunity to expand and proliferate? Perhaps valuing the complex interlinkages of located environmental changes and varied articulations of (scientific) labour in the knowledge making process is one place to start.

## Data Availability

The data that support the findings of this study are available from the corresponding author upon reasonable request.

## Footnotes

1 There is a long history of tension between local fishers and researchers, who largely came from elsewhere in the Netherlands and Belgium (and later, the world). Fishers generally feel threatened by researchers who they fear will restrict access to their livelihood. In the case of the Eastern Scheldt, these oppositions and affiliations ebb and flow over time as actors’ matters of concern converge or come to oppose one another, much as Callon describes in his famous essay (Callon, 1984).

2 The Dutch have a long history attempting to keep the North Sea at bay. In recent years, emerging approaches attempt to harness the power of natural processes, generally referred to as *Bouwen met Natuur* (tr. ‘Building with Nature’, see De Vriend et al., 2014). This project seeks to exploit the capacities of the ecosystem to keep sediments along the coast, for example by encouraging the growth of salt marsh plants that are able to retain sediments in their roots. A more extreme example is the geoengineering project known as the *Zandmotor* (tr. Sand Engine)*,* through which an enormous sand enrichment relies on wind, tides, and currents to slowly reinforce the Dutch coastline (see Helmreich, 2023).

3 Scholars of science and technology have long considered the role of non-epistemic values in making scientific knowledge, for example in terms of trade-offs between different values (Elliott & McKaughan, 2014). Our notion of brackish knowledge instead emphasizes the layered interplays among material, epistemic, and institutional conditions; these layers can align or clash without reducing to a single zero-sum trade-off.

4 Global and regional models do not nest neatly inside one another. However, the output of global models often informs the boundary conditions for regional and small-scale models, meaning they provide numerical data to the model code about what the environment is like on the borders around the geographical area in question, such as how deep the water is or what temperature the water is. These data then provide a starting point for the model’s calculations.

5 Although model validation often relies on comparisons to existing observational data, there is not strict directionality from observation to modelling. Indeed in some cases models are able to identify errors in observational data, by for example pinpointing irregularities that are later identified as the result of faulty measurement instruments. See Edwards (2010) for further discussion on this subject.
